# Displacement of the Epiglottis

**Published:** 1851-04

**Authors:** Jas. Kitsell


### ARTICLE XXII.

*Displacement of the Epiglottis.* By Jas. Kitsell, Esq.

John Chillinsworth, aged 72, now an inmate of the Droitwich Union House, has, for the last six months, labored under painful difficulty of deglutition, being unable to swallow any solid food, which inconvenience occurred during violent and repeated attacks of retching and vomiting. On examination, the epiglottis may be readily seen projecting upwards at the base of the tongue, as though it was almost detached from its connection with the glottis. In consequence of the patient's advanced age, peculiar idiosyncrasy, and irritable temperament, no attempt has been made to replace the epiglottis, especially as the spasmodic symptoms are much abated, although any attempt to swallow solid food produces spasmodic affection of the part, whereby it is forcibly rejected, but the parts have in a great measure adapted themselves to the inconvenience, and he can swallow liquids tolerably well. In the event of death, I hope to avail myself of a *post-mortem* examination to ascertain the real cause of this displacement.—*Prov. Med. & Surg. Jour.*

